# Mesenchymal Stem Cells of Dental Origin for Inducing Tissue Regeneration in Periodontitis: A Mini-Review

**DOI:** 10.3390/ijms19040944

**Published:** 2018-03-22

**Authors:** Beatriz Hernández-Monjaraz, Edelmiro Santiago-Osorio, Alberto Monroy-García, Edgar Ledesma-Martínez, Víctor Manuel Mendoza-Núñez

**Affiliations:** 1 Research Unit on Gerontology, FES Zaragoza, National Autonomous University of Mexico, 09230 Mexico City, Mexico; beatrizhmonjaraz@hotmail.com; 2 Haematopoiesis and Leukaemia Laboratory, Research Unit on Cell Differentiation and Cancer, FES Zaragoza, National Autonomous University of Mexico, 09230 Mexico City, Mexico; edelmiro@unam.mx(E.S.-O.); 2814.260@gmail.com(E.L.-M.); 3 Immunology and Cancer Laboratory, Oncology Research Unit, Oncology Hospital, National Medical Center, IMSS, 09230 Mexico City, Mexico; albertomon@yahoo.com

**Keywords:** DPSC, biological mechanism, periodontal treatment

## Abstract

Periodontitis is a chronic disease that begins with a period of inflammation of the supporting tissues of the teeth table and then progresses, destroying the tissues until loss of the teeth occurs. The restoration of the damaged dental support apparatus is an extremely complex process due to the regeneration of the cementum, the periodontal ligament, and the alveolar bone. Conventional treatment relies on synthetic materials that fill defects and replace lost dental tissue, but these approaches are not substitutes for a real regeneration of tissue. To address this, there are several approaches to tissue engineering for regenerative dentistry, among them, the use of stem cells. Mesenchymal stem cells (MSC) can be obtained from various sources of adult tissues, such as bone marrow, adipose tissue, skin, and tissues of the orofacial area. MSC of dental origin, such as those found in the bone marrow, have immunosuppressive and immunotolerant properties, multipotency, high proliferation rates, and the capacity for tissue repair. However, they are poorly used as sources of tissue for therapeutic purposes. Their accessibility makes them an attractive source of mesenchymal stem cells, so this review describes the field of dental stem cell research and proposes a potential mechanism involved in periodontal tissue regeneration induced by dental MSC.

## 1. Introduction

Periodontitis is a chronic inflammatory disease of the supportive tissues of the teeth. This disease is caused by specific microorganisms or groups of specific microorganisms, which result in a pathological disinsertion of the collagen fibres of the cementum; progressive destruction of the periodontal ligament and alveolar bone with increased probing depth formation, recession, or both; and apical migration of the union epithelium [1].

When these conditions last over time, they cause the tissue to continue to be destroyed until the tooth is lost due to lack of support. This not only has repercussions at the local level that affect the chewing, phonation, and aesthetics of the patient but it is also related to other pathologies that affect quality of life [2].

Although the disease can be treated successfully in its early stages, unfortunately, it is diagnosed when it affects the periodontal ligament, which causes most patients to seek dental care when the disease is very advanced, and the chances of keeping the tooth in the mouth are minimal. Consequently, different therapeutic options focus on recovering the lost health of the tissues (alveolar bone, periodontal ligament, and cementum). The conventional treatment consists of emphasizing hygiene, performing scaling and root planing, providing antibiotics, and, occasionally, performing flap surgery to access the root surfaces to debride them properly [3].

These actions stop the acute phase of the disease, and sometimes a significant amount of new connective tissue insertion is recovered; however, the regeneration of the complex structure of the periodontium is not achieved. Conventional treatment relies on natural and synthetic materials that fill defects and replace lost dental tissue, but these approaches are not substitutes for a real regeneration of tissue with a physiological architecture and function. To address this, there are successful engineering initiatives. The purpose of tissue engineering is the regeneration of tissues through the combined use of biomaterials and biologic mediators in order to create living tissues that can replace structures or functions that have been lost. In this sense, there are several approaches to tissue engineering for regenerative dentistry, among them, the use of stem cells. In particular, stem cells have great versatility at the level of tissue regeneration for many different characteristics and can modulate chronic inflammation, a central feature in periodontitis. Given the characteristics of these cells, they are considered a potentially useful tool for efficient regeneration of periodontal tissues [4].

Therefore, this mini-review presents the basic concepts of periodontitis, the mesenchymal stem cells of dental origin that are used in the treatment, and the possible molecular mechanism involved in the regeneration of periodontal tissues.

### 1.1. Periodontitis

Periodontitis is a condition that destroys the tissues around the tooth, which evolves to tooth loss and triggers various complications at the local and systemic level [5,6].

Periodontitis is one of the diseases in which the importance of an imbalance of species of pathogenic and beneficial microorganisms is well demonstrated. Dysbiosis in the initial stage is generally induced by poor hygiene that causes the accumulation of different types of bacteria such as *Aggregatibacter actinomycetemcomitans* (*A. actinomycetemcomitans*) and, specifically, those of the red complex: *Porphyromonas gingivalis* (*P. gingivalis*), *Tannerella forsythia* (*T. forsythia*), and *Treponema denticola* [7].

**Figure 1:**
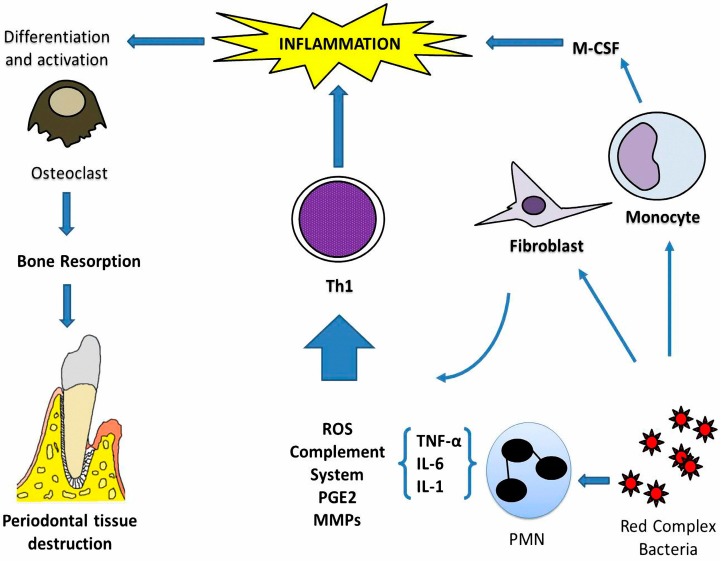
Pathophysiological mechanisms in periodontitis. The presence of red complex bacteria promotes periodontal inflammation in susceptible individuals. Activated polymorphonuclear neutrophils (PMN), fibroblast, and monocytes in the oral cavity induce production of cytokines such as tumour necrosis factor α (TNF-α), interleukin (IL)-1, and IL-6. The initial function of this inflammation is to protect against bacteria; however, chronic inflammation induces enhanced reactive oxygen species (ROS), complement system, and PGE2 and matrix metalloproteinases (MMPs) such as gelatinase B and collagenase 1. This inflammatory microenvironment induces a Th1 lymphocyte profile, which promotes inflammation and is associated with the maintenance and progression of the lesion. In addition, activated monocytes induce cytokines as M-CSF (macrophage colony-stimulating factor) that promote activation and differentiation of osteoclasts, which are related to resorption of alveolar bone, damage to cementum, and periodontal ligament.

Although periodontitis is initiated by an imbalance that causes the accumulation of these bacteria and their lipopolysaccharides (LPS), the destruction of the supporting tissues of the tooth is mainly due to an exacerbated immune response of the host in susceptible individuals, which prevents the acute inflammation from being effectively resolved and initiates chronic periodontitis [8]. (Figure 1). In these cases, the accumulation of bacteria in the gingival sulcus causes the migration of polymorphonuclear neutrophils (PMNs) and monocytes. These cells, together with those of the gingival epithelium, secrete cytokines such as interleukin (IL)-1β, IL-6, tumour necrosis factor α (TNF-α), and adhesion molecules such as endoglin and intercellular adhesion molecule 1 (ICAM-1), which increase the adhesion of PMNs and monocytes to endothelial cells and increase the permeability of the gingival capillaries, which leads to the accumulation of leukocytes in the infection zone [9].

This allows the macrophages that have arrived at the area of the lesion to produce prostaglandin 2 (PGE2). High levels of this molecule and IL-1β increase the binding of PMNs and monocytes to endothelial cells, exacerbating inflammation, which, together with IL-6 and TNF-α, induce osteoclasts to activate and reabsorb the alveolar bone [10,11].

Meanwhile, local capillaries release a large amount of serum as a result of the release of histamine and complement molecules, which leads to increased vascular permeability. This serum is converted into a tissue fluid that contains inflammatory peptides (antibodies, complement, and other agents that mediate the body’s defence) that are carried into the gingival sulcus. Increased gingival fluid causes the tissues and the amount of gingival crevicular fluid to increase in volume [11]. Macrophages and neutrophils in the infection area contain enzymes such as nicotinamide adenine dinucleotide phosphate (NADPH) oxidase and myeloperoxidase that produce reactive oxygen species (ROS) to eliminate pathogens [12,13].

Under normal conditions, antioxidant mechanisms protect the tissues from damage mediated by ROS. However, if the body’s antioxidant capacity is insufficient against ROS, oxidative stress (OxS) occurs that damages the hard and soft tissues of the periodontium [14,15].

OxS causes oxidation of important enzymes, stimulation of release of more proinflammatory cytokines, lipid peroxidation, and damage to DNA and proteins. These mechanisms affect the gingival tissues, periodontal ligament, and alveolar bone that support the teeth [16,17]. In addition, excessive release of pro-inflammatory cytokines is stimulated through the activation of nuclear factor κβ (NF-κB) and the production of PGE2 through lipid peroxidation and superoxide release, which is related to bone resorption [18].

If this situation is sustained, the epithelial adhesion is destroyed, and the alveolar crest loses its height, which translates clinically into dental mobility and formation of periodontal pockets, causing the accumulation of more bacteria that increase the problem, thereby completely destroying the periodontal ligament; the alveolar bone becomes atrophied, and the tooth is lost [19,20].

To avoid this outcome, conventional treatment for periodontitis patients is divided into three different phases, which often overlap. The initial phase is focused on stopping the progression of destruction of periodontal tissues by eliminating local factors through oral hygiene instructions combined with scaling and root planing. The second phase is corrective and is aimed at restoring the function and aesthetics of tissues, while the last phase is considered periodontal maintenance that is intended to prevent the recurrence of periodontitis [21].

Even when this treatment is carried out with rigor, the results are mostly aimed at the stabilization of the disease and not the regeneration of the lost periodontal tissues [22]. Therefore, it is necessary to carry out other procedures to recover tissue insertion, including root surface conditioning, bone grafting, guided tissue regeneration, and the application of growth factors [21].

Despite the use of these treatments, the original anatomy and physiology has not been restored, and in some cases, periodontal aberrations such as ankylosis, gingival recession, and formation of compact bone have developed [23,24]. Therefore, new therapeutic options, such as the use of mesenchymal stem cells (MSCs), including those isolated from dental tissues, have been proposed [25,26].

### 1.2. Mesenchymal Stem Cells

**Figure 2:**
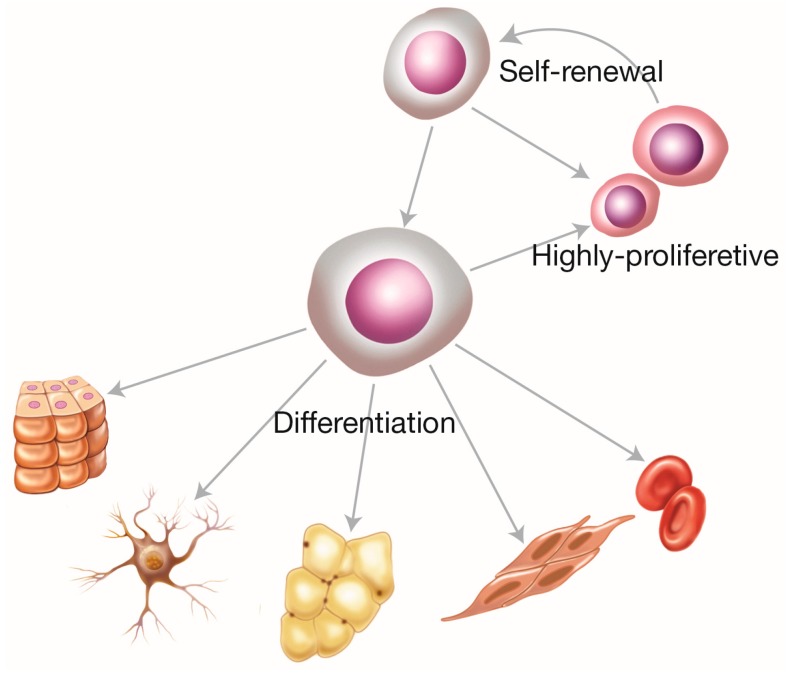
Characteristics of mesenchymal stem cells. The scheme shows the cellular differentiation capacity considering autoregeneration, replication, and differentiation in epithelial, neural, adipose, muscular, and sanguine cells.

Mesenchymal stem cells (MSCs) are adult connective tissue cells that originate from the mesoderm. A stem cell is an undifferentiated cell that is distinguished by its capacity for autoregeneration (which perpetuates the population of stem cells) and by its capacity for asymmetric division, which indicates a daughter stem cell and another with greater commitment to proliferation and cell differentiation [27,28] (Figure 2).

Stem cells can be isolated in the early stages of embryogenesis (embryonic stem cells) or in various postnatal tissues (adult stem cells). Although embryonic stem cells have a long lifespan and potential for high differentiation, the bioethical aspects involved in their production have focused the research on adult stem cells, which are considered pluripotent and found in most tissues, with the potential to repair damaged tissues or renew cell populations that are constantly being replaced [29,30,31,32].

MSCs are involved in growth, wound healing, and replacement of cells that are lost daily by exfoliation or in pathological conditions. Different studies have shown that they induce repair in neuronal, hepatic, and skeletal muscle after infusion in both preclinical and clinical models [33,34,35]. These qualities make them a potential tool for tissue engineering and tissue repair [36]. Interestingly, it has been shown that allogeneic MSC infusions are well tolerated, and there appear to be no side effects (acute or late) and no ectopic tissue formation [37].

**Figure 3:**
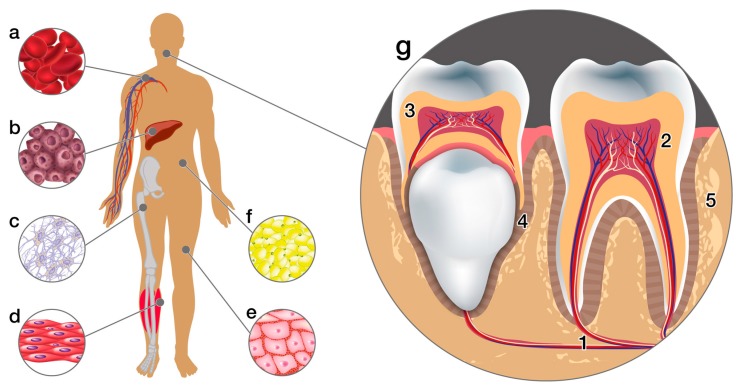
Sources of stem cells in the human organism. The diagram shows some tissue sources of adult stem cells. (**a**) Peripheral blood, (**b**) Liver, (**c**) Bone marrow, (**d**) Muscles, (**e**) Skin, (**f**) Adipose tissue, (**g**) Dental tissues: (1. Apical dental papilla, 2. Adult pulp, 3. Pulp of deciduous teeth, 4. Periodontal ligament, and 5. Alveolar bone).

Another advantage of MSCs is that they can be obtained from various sources of adult tissues such as bone marrow, adipose tissue, skin, and tissues of the orofacial area [38,39,40,41,42,43,44]. The first cells isolated from the orofacial area were those of third molar dental pulp. Subsequently, MSCs have been isolated from deciduous teeth, apical papilla, and periodontal ligament [45,46,47]. (Figure 3).

### 1.3. Mesenchymal Stem Cells of Orofacial Area

Within the orofacial area, there are several sources of MSCs, including periodontal ligament stem cells (PL-MSC), apical papilla derived stem cells (SCAP), dental follicle cells (DFC), and dental pulp mesenchymal stem cells (DP-MSC) from both deciduous (SHED) and permanent teeth (DPSC). They all have attracted scientific interest, given their similarity to MSCs derived from bone marrow, their immunoregulatory capacity, and their minimally invasive obtention procedure [48,49].

The human periodontal ligament (PDL), a fibrous connective tissue that surrounds and supports the tooth, contains a small subpopulation of MSC that is responsible for maintaining and regenerating periodontal tissue structure and function. These cells form osteoblasts, fibroblasts, and tooth cementoblasts that form cementum- and periodontal ligament-like tissues [47].

SCAP and DFC are stem cells that are located only in the developing tooth germ before they erupt into the oral cavity; SCAP is at the tips of growing tooth, and DFC is in a connective tissue sac surrounding the enamel organ and dental papilla. These cells form adherent clonogenic clusters and similar to other MSC populations; they differentiate into adipocytes and odontoblasts/osteoblasts [50], progenitors for cementoblasts, and periodontal ligament (PL) [51]. DFC are believed to be parent cells that differentiate into PDL fibroblasts, osteoblasts, and cementum-fabricating cementoblasts during the development of the periodontium [52].

DP-MSC are cells in the pulp chamber of the tooth that originate from the cranial neural crest. They have clonogenic capacity and rapid proliferative rates, can differentiate into various cell types such as chondrocytes, adipocytes, and odontoblasts, and can even be reprogrammed into myocytes or neurons. Similar to bone marrow, the type of surface markers they express are Stro-1, CD29, CD73, CD90, CD105, and CD166, and they are negative for haematopoietic markers such as CD14, CD45, CD34, CD25, and CD28 [53].

The differentiation of DP-MSCs and PL-MSC into the different cell strains is determined by the microenvironment surrounding the cells, such as growth and transcription factors, molecular receptors, and extracellular matrix proteins. In addition, both in vitro and in vivo studies have shown that both have the ability to form mineralized tissues [54,55,56,57,58,59].

### 1.4. MSCs of Dental Origin in Periodontal Regeneration

One of the advantages of MSCs of dental origin is that they can be safely used in allogeneic transplants, because they have immunosuppressive properties such as those found in bone marrow-derived cells [60]. Good immune tolerance and tissue repair ability have both been observed in different models. Also, it is necessary to evaluate the effectiveness of MSC, DP-MSC, SCAP, and PL-MSC to form dental tissues and regenerate lost periodontium in several animal and human models [61] (Table 1).

In addition to their immunomodulatory function, they also have an anti-inflammatory function, which suggests that they can stop the development of lesions and allow the intrinsic regenerative processes to have a greater possibility of success. In this sense, MSCs of dental origin may have a direct or indirect effect on the control of OxS processes and the inflammation characteristic of periodontitis, since it has been suggested that these cells have an anti-inflammatory effect that could help to reduce the high levels of ROS and cytokines that are present in periodontitis [62].

These cells have demonstrated a strong ability to induce bone formation in vivo. According to investigations, DP-MSCs of exfoliated teeth cannot differentiate directly into osteoblasts but may induce the formation of new bone by forming an osteoinductive template to recruit osteogenic cells from the host [45]. As DP-MSCs are common progenitors of odontoblasts and osteoblasts, they are considered useful for periodontal tissue engineering [63,64]. In addition, MSCs may exert a neovascularization effect; therefore, one of the therapeutic functions is the early induction of granulation tissue, which, in the case of periodontitis, will be followed by stabilization of the neovascular network in the periodontal niche [65].

On the one hand, histological findings in animal models suggest that the application of MSCs of dental origin can promote the regeneration of infra-bone defects. Rats with bone defects that were treated with PL-MSCs presented a higher percentage of bone-type filler after surgery, as well as the generation of structures similar to cementum and periodontal ligament after 21 days of treatment [66]. Similarly, Yu et al. observed in a rat model that the placement of PL-MSCs on a gelatinous scaffold is effective at achieving the regeneration of soft and hard periodontal tissues [67].

On the other hand, a study that aimed to understand the effect of DP-MSCs in a preclinical miniature pig model demonstrated that allogeneic transplantation of DP-MSCs from deciduous teeth can effectively repair the loss of hard and soft tissue caused by periodontitis without adverse effects [60].

To accelerate the repair of artificially created dehiscence to emulate the tissue destruction of patients with advanced periodontitis, a study was conducted in sheep in which PL-MSCs were placed in a collagen scaffold. The results showed extensive coverage of new alveolar bone with a cell line similar to osteoblasts at 4 weeks of treatment [68].

On the other hand, it has been observed that a functional connection between the cementum, ligament, and alveolar bone requires cells, a series of chemical signals and a physical support called scaffolding. In this regard, it has been shown that DP-MSCs promoted regeneration in periodontal defects when placed in combination with biomaterials [69].

Likewise, it has been found that DP-MSCs placed in a collagen scaffold can be used to repair bone defects in humans, since this allograft produces rapid regeneration of alveolar bone in both quality and quantity of tissue compared with techniques that use bone from various sources for guided regeneration [70].

In this sense, Aimetti et al. described a clinical case of a periodontal defect in a 56-year-old male treated with DP-MSCs obtained from one of his third molars. At one year after surgery, it was observed clinically and radiologically that the defect had been completely filled by bone-like tissue [71].

### 1.5. Biological Mechanisms of MSCs of Dental Origin Involved in Periodontal Regeneration

**Figure 4:**
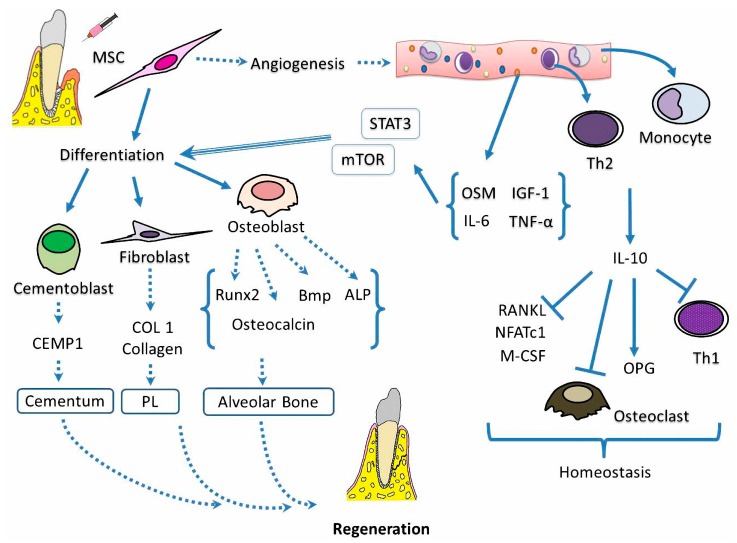
Putative mechanism involved in the regeneration of periodontal tissue via DP-MSCs. The placement of DP-MSCs allows the neoformation of blood vessels that carry signals (IL-6, IGF-1, and TNF-α andoncostatin M: OSM) and cells (Th2 and monocytes) to the site of the lesion. Signals, via the STAT3 and mTOR pathways, will allow the DP-MSCs to be transformed into osteoblasts, which in turn will produce ligands to bone morphogenic protein 2 (BMP), alkaline phosphatase (ALP), and runt-related transcription factor 2 (Runx2) for the generation of alveolar bone, whereas the production of IL-10 and osteoprotegerin (OPG) will create a suitable microenvironment in which osteoclast activity is inhibited and tissue repair is promoted. On the other hand, DP-MSCs also differentiate into cementoblasts and fibroblast, which secrete proteins such as CEMP1 and COL1 for synthesis of cementum and collagen fibres of the periodontal ligament, respectively. Finally, the neoformation of blood vessels, an environment with the indicated signals and the differentiation of DP-MSCs into the constituent cells of the periodontium, will jointly achieve the regeneration of periodontal tissues.PL: Periodontal ligament; Bmp: Bone morphogenic protein; RANKL: Receptor activator of nuclear factor kappa-Β ligand; NFATc1: Nuclear factor of activated T-cells, cytoplasmic 1; M-CSF: Macrophage colony-stimulating factor.

For the transplantation of dental origin MSCs to regenerate injured periodontium, it is essential to have at least three factors: blood supply, adequate molecular signals and proliferation, and differentiation towards the cellular precursors that are capable of regenerating the lost tissues. (Figure 4).

During the formation of new periodontal tissues, it is necessary to have sufficient blood supply, because the molecules that are necessary for regeneration will arrive through vessels. DP-MSCs can exert a neovascularization effect that induces early granulation tissue, which, in the case of periodontitis, is followed by the formation of a vascular network in the periodontal niche [65,87]. 

There is even evidence that after transplantation of DP-MSCs from deciduous teeth, they can differentiate into functional blood vessels that connect to the recipient vessels [88].

To have a suitable means for DP-MSC differentiation, it is necessary to have signals that arrive through neoformed blood vessels such as insulin-like growth factor 1 (IGF-1), vascular endothelial growth factor (VEGF), transforming growth factor β1 (TGF-β1), and hepatocyte growth factor (HGF) [89], which help to reduce inflammation and induce the regeneration of periodontal tissue. In addition, it is known that adequate amounts of TNF-α and IGF-1 are generated after the surgical procedure for the placement of cells, which contribute to regeneration [90].

TNF-α has no direct effect on the proliferation or cell cycle of DP-MSCs; however, it increases the mineralization of the extracellular matrix and the expression of mineralization-related genes such as bone morphogenic protein 2 (BMP2), alkaline phosphatase (ALP), runt-related transcription factor 2 (Runx2), and collagen I (COL-I) via the NF-κB pathway during osteogenic differentiation [91,92].

On the other hand, sufficient amounts of IGF-1 promote collagen synthesis in fibroblasts and differentiation into osteoblasts via the mTOR pathway and stimulate Runx2 and the production of osteocalcin [93]. Osteocalcin is also stimulated by IL-6. The presence of osteocalcin induces the production BMP2 and Runx2 ligands for the synthesis of extracellular matrix by osteoblasts [56].

Thus, while stimulating the synthesis of an extracellular matrix that is later to be mineralized is one factor, there are also others that play a crucial role in periodontal regeneration such as extracellular matrix metalloproteinases (MMPs) and their inhibitors (TIMPs) [94]. MSC delivery has been shown to reduce the expression of MMP-1 and MMP-8 but to increase that of TIMP-2 and TIMP-4, whereby it is possible that MSC placement allows an optimal state of remodelling of the extracellular matrix during osteogenic differentiation [95].

On the other hand, both monocytes and Th2 lymphocytes that travel in the bloodstream produce IL-10, which in turn favours the expression of osteoprotegerin (OPG) and negatively regulates RANKL, NFATc1, and M-CSF, thereby decreasing differentiation and activation of osteoclasts by reducing bone resorption. At the same time, the inflammatory environment caused by Th1 is reduced, and the expression of Th2 and the anti-inflammatory microenvironment are favoured [96].

The third element to be taken into account is the DP-MSCs that are placed in the damaged periodontal tissue, which can proliferate and differentiate into the precursors of cementoblasts, osteoblasts, and fibroblasts that are responsible for the generation of alveolar bone, root cementum, and periodontal ligament, respectively.

Oncostatin M (OSM), which is a cytokine of the IL-6 family, as well as IL-6 itself, induces differentiation into preosteoblasts, since IL-6 is related to the production of ALP, which is required for the formation of osteogenic nodules, promotes the mineralization of the extracellular matrix, and increases the expression of genes associated with osteogenesis using the JAK3/STAT3 signalling pathway [97,98,99].

In an in vivo analysis of tissue formation, it was shown that the DP-MSCs from permanent and deciduous teeth can differentiate and form bone [100]. 

Finally, it has been observed that DP-MSCs differentiate into cementoblasts and fibroblasts, which, respectively, secrete the CEMP-1 protein and COL-1 for the synthesis of radicular cementum and collagen fibres of the periodontal ligament, respectively. In this way, the three elements contribute to functional periodontal regeneration [101]. (Figure 4).

### 1.6. Current Challenges in MSC Dental Origin Use

Research on mesenchymal bone marrow stem cells is a widely explored field of knowledge; however, those of dental origin are in an incipient stage. In this sense, it could be argued that we still lack the requisite biological understanding of these cells to apply them to a human patient. Even something as elementary as the choice of differentiation markers that would allow a unified international understanding of the use of MSC of dental origin is a recurrent but elusive theme in research. However, this does not seem to impede the race to position MSCs as an alternative therapy.

Indeed, along with cutting-edge clinical trials, we continue to address issues that are related to the supply and procurement of enough cells for therapeutic purposes, their senescence prevention, and their guaranteed proliferation and expansion without detriment to their ability to differentiate. Beyond the questions of basic science, which will undoubtedly have to be resolved in the future, there are still issues that are related to in vivo functionality. Studies of xenotrasplant have shown that it is possible to form complex dentin, root cementum, periodontal ligament, tissues such as bone, etc. By transplanting MSCs of dental origin in various animal models, we have clear evidence that these stem cells have the potential to induce the formation of complex structures such as dental tissues. (Table 1). However, these experimental animal data provided only an approximation of the anticipated behaviour in a human being. Clinical trials have not been widely reported and, as expected, at this early stage of their study, core issues regarding threats to human health and related questions need to be discussed. 

The available human studies suggest that there are no adverse effects or ethical implications, but there are still very few of them that are needed to reach definitive conclusions. The history of medicine has taught us that an emerging procedure can rise to triumph only to eclipse later due to the development of unacceptable side effects [102]. This issue is exacerbated if the potential risk of the developing procedure exceeds its benefits. In this sense, the use of MSC of dental origin for the reconstruction of dental tissues could seem precipitous in the context of periodontitis, because it does not intrinsically endanger the life of the patient, and there are alternative procedures that are already widely known. However, despite the advances in current infections, this problem is closely associated with the loss of teeth. Therefore, the search for new therapeutic trends is still valid, and one proposal is the use of mesenchymal stem cells.

## 2. Conclusions

Biological, molecular, and clinical evidence supports the potential use of MSCs as an alternative therapy in diverse pathologies. In this sense, the use of MSCs of dental origin to repair and regenerate periodontal tissues can offer advantages over other more exploited cellular sources. Because of its similarity to target tissue and remarkable accessibility, it seems reasonable to conclude that using MSCs of dental origin to treat periodontitis is the most logical option. In addition, it would entail the least risk, since the therapeutic cells come from the same tissue that is intended to be cured, which would not be the case of MSCs that originated from bone marrow or adipose tissue. While this assumption needs to be verified in future studies, it does not, however, diminish the prospects for therapeutic use of MSC.

## Figures and Tables

**Table 1:** Studies on the application of dental stem cells in the repair of bone defects caused by periodontitis.

Cell Type	Receiver	Objective	Findings	Source
DP-MSC	Immune-compromised mice	To know the capacity to form bone	Cultured human DP-MSC produced calcified tissue that was histologically proved to be bone when transplanted into immunocompromised mice.	[63]
DPSC	Swine	To investigate the roles of the hepatocyte growth factor (HGF) and DPSCs in periodontal tissue regeneration	Cells and HGF that were produced significantly improved periodontal bone regeneration in swine.	[72]
DPSC	Immune-compromised mice	Compare DPSC with bone marrow cells in the formation of the dentin-pulp complex	The transplanted cells generated a dentine structure covered with odontoblasts surrounding pulp tissue. The dentin-pulp complex was formed from dental pulp stem cells.	[48]
DPSC	Humans	Demonstrate that the biocomplex of DPSC and collagen sponge can be used to repair bone defects in humans	Autografts produced rapid bone regeneration, which was of optimum quality and quantity compared to standard techniques for guided regeneration.	[70]
DPSC	Humans	To regenerate the infrabony defect on the mandibular right second premolar	The defect was filled with bonelike tissue, as confirmed through the reentry procedure.	[71]
SHED	Immune-compromised mice	To know the characteristics and potential of development in vivo	SHED was able to differentiate into odontoblasts and induce osteoblasts to form bone in vivo; however, they were unable to regenerate the dentin-pulp complex.	[45]
SHED	Swine	To investigate the ability of allogeneic SHEDs to regenerate lost periodontium in a swine periodontitis model	The effective repair of the loss of hard and soft tissue caused by periodontitis was observed.	[60]
PL-MSC	Immune-compromised mice	To know the spatial distribution of the stem cells in the periodontal ligament	The stem cells found on the alveolar bone had a greater potential for multilineage differentiation than those found on the root surface, both in osteogenic and adipogenic differentiation.	[73]
PL-MSC	Dog	Examine stem cells derived from multiple layers of ligament for periodontal regeneration	Cell formation was observed on the defect walls with periodontal ligament and polyglycolic acid stem cells.	[74]
PL-MSC	Dog	To know the ideal cell type for clinical application	Stem cells provided incremental lines of neo cement, with Sharpey fibres being inserted and cellular cementum at the apex of the root.	[75]
PL-MSC	Dog	To histomorphometrically evaluate the use in the treatment of class III furcation defects	Ligament cells, in association with guided tissue regeneration, were able to significantly promote periodontal regeneration.	[76]
PL-MSC	Swine	To explore the potential of using autologous periodontal ligament stem cells to treat periodontal defects	Stem cells were effective in autologous transplantation, which was used to treat periodontitis in a preclinical miniature swine model.	[77]
PL-MSC	Swine	To develop a feasible allogeneic cell-based method for the treatment of periodontitis	Allogenic stem cells were able to repair bone defects in an experimental model of periodontitis without immunological rejections.	[78]
PL-MSC	Swine	To evaluate the bone regeneration potential of biomimetic intrafibrillarly mineralized collagen (IMC) loaded with autologous periodontal ligament stem cells (PL-MSC s) in large bone defects	IMC achieved a significantly higher extent of forming new bones, with the normal architecture of natural bones and blood vessels.	[79]
PL-MSC	Humans	To know the utility of autologous progenitor cell transplantation in tissue repair	It was shown that transplantation of autologous periodontal ligament progenitor cells was able to provide a therapeutic benefit in periodontal defects.	[80]
PL-MSC	Humans	To evaluate the safety of autologous transplantation and its effectiveness as adjuvant to graft materials in the repair of bone defects caused by periodontitis	The use of stem cells did not produce adverse effects but was effective at repairing bone defects.	[81]
PL-MSC	Immune-compromised mice	Recreate a favourable regeneration microenvironment and enhance the reconstruction of physiologic architecture of a dental cementum/PDL-like complex	The mixed-type PL-MSC pellets supported cementum/periodontal ligament (PDL)-like tissue regeneration with neovascularization.	[82]
SCAP	Immune-compromised mice	Evaluate the potential application of these cells for cementum/PL regeneration and bio-root engineering	Tissue-regenerative capacity was shown to produce a typical cementum/PDL-like complex in vivo.	[83]
DFC and PL-MSC	Immune-compromised mice	To evaluate DFCs that could enhance the function of both PL-MSCs by providing a beneficial young microenvironment	PL-MSCs co-cultured with DFCs produced a typically arranged tissue with Sharpey-like perpendicular fibres. Additionally, a root/periodontal ligament-like complex and a periodontal ligament/bone-like complex were observed.	[84]
DP, SHED and PL-MSC	Immune-compromised mice	To evaluate the effectiveness of MSC to form dental tissues	DPSC and SHED were able to generate a dentin-pulp complex. PL-MSC generated structures associated with the periodontium.	[85]
CSC and PL-MSC	Dog	To evaluate the regenerative potential in experimentally created periodontal intrabony defects	Higher amounts of new cementum were formed and a larger dimension of new connective tissue. Cellular therapy promoted periodontal regeneration in experimental intrabony periodontal defects.	[86]

Dental Pulp Mesenchymal Stem Cells (DP-MSC), third molar human dental pulp stem cells (DPSC), stem cells from human exfoliated deciduous teeth (SHED), periodontal ligament stem cells (PL-MSC), cementum stem cells (CSC), root apical papilla derived stem cells (SCAP), dental follicle cells (DFC).

## Abbreviations

ALP	Alkaline phosphatase
BMP2	Bone morphogenic protein 2
CSC	Cementum stem cells
COL-I	Collagen I
DFC	Dental follicle cells
DP-MSC	Dental pulp mesenchymal stem cells
DPSC	Permanent teeth stem cells
HGF	Hepatocyte growth factor
IL	Interleukin
ICAM-1	Intercellular adhesion molecule 1
IGF-1	Insulin-like growth factor 1
IMC	Intrafibrillarly-mineralized collagen
LPS	Lipopolysaccharides
M-CSF	Macrophage colony-stimulating factor
MMPs	Matrix metalloproteinases
MSCs	Mesenchymal stem cells
NF-kB	Nuclear factor kappa beta
OPG	Osteoprotegerin
OSM	Oncostatin M
OxS	Oxidative stress
PL-MSC	Periodontal ligament mesenchymal stem cells
PGE2	Produce prostaglandin 2
PL	Periodontal ligament
PMNs	Polymorphonuclear neutrophils
ROS	Reactive oxygen species
Runx2	Runt-related transcription factor 2
SCAP	Stem cells from root apical papilla derived
SHED	Stem cells from both deciduous teeth
TNF-α	Tumour necrosis factor alpha
TGF-β1	Transforming growth factor beta 1
TIMPs	Tissue inhibitor of metalloproteinases
VEGF	Vascular endothelial growth factor
